# Editorial: From mechanosensing to signalling and cell response: The ion channel force

**DOI:** 10.3389/fcell.2023.1183726

**Published:** 2023-03-27

**Authors:** Dimitra Gkika, Albrecht Schwab

**Affiliations:** ^1^ University Lille, CNRS, Inserm, CHU Lille, UMR9020-U1277 -CANTHER -Cancer Heterogeneity Plasticity and Resistance to Therapies, Lille, France; ^2^ Institute of Physiology II, University of Münster, Münster, Germany

**Keywords:** piezo, force-from-tethering gating, cancer microenvironment, osteoarthritis, macrophage, dendritic cell, TRP

The cellular microenvironment is full of mechanical challenges ranging from shear and compressive stress to tension and substrate stiffness. Ion channels can sense all these mechanical stimuli directly through innate force sensing or through lipids and filaments, including cytoskeleton and extracellular matrix. Mechanical forces are therefore rapidly transformed in electrochemical signals through the activity of ion channels which in turn defines cellular physiology and fate. Mechanosensitive ion channels (MIC) are essential in controlling a variety of cellular processes such as transcription, differentiation, adhesion and migration, and their multifaceted consequences in pathophysiology: hearing, touch, neurotransmission, development, hemodynamics, inflammation, neurodegeneration, cardiovascular disease and oncogenesis. This Research Topic presents some recent advances on the molecular mechanisms of MIC activation and regulation as well as their effects on intracellular signaling and cellular responses. The Research Topic comprises four Review papers going from the mechanistic aspect of the MIC as a mediator of mechanical communication between the cell cytoskeleton with extracellular matrix in all cell types (Chuang and Chen) to specific cell type MIC responses in immune cells (Lee et al.), chondrocytes (Gao et al.) and cancer cells (Bera et al.).

Mechanosensing is not limited to specialized sensory cells but is present in all kinds of cell types which transform the mechanical forces into ionic currents. Two main models have been described to explain MIC gating: The membrane tension model for which the force to open the MIC comes from the lipid bilayer tension; in the second model the force coming from the extracellular matrix is transmitted through a tether connecting the MIC with the cytoskeleton. Chuang and Chen give an overview of the studies supporting force-from-tethering gating found in different organisms with a focus on Piezo and DEG/ENaC/ASIC and TRP ion channel families. Of particular interest are the tools such the micropipette-guided ultrasound stimulation as well as new biophysical approaches and the question on how the tethering force transmits the stress to gate MIC opening.

Immune cell signaling is usually associated with chemical cues originating, for example, from the inflammatory microenvironment. However, there is increasing evidence that immune cells also respond to mechanical cues from their environment. Lee et al. provide an overview of the emerging field of immune cell mechanotransduction. They focus on macrophage and dendritic cell behavior and their regulation by TRPV4 and Piezo1 channels.

Articular chondrocytes are exposed to a particularly challenging mechanical input. Thus, it does not come as a surprise that these cells are well equipped with mechanosensitive Ca^2+^-permeating channels that trigger force-dependent cartilage remodeling and injury responses. Gao et al. discuss the current understanding of the roles of Piezo1, Piezo2, and TRPV4 in cartilage health and disease. They also discuss whether these channels could be targeted in order to prevent cartilage degeneration associated with osteoarthritis.

During the different steps of the metastatic cascade, cancer cells face various physical cues by both solid (extracellular matrix stiffness and viscoelasticity, mechanical compression, solid barriers, confined tracks) and fluid (fluid viscosity, hydraulic resistance, interstitial fluid pressure, turbulent flow) surroundings of the tumor microenvironment. Bera and others explain in the first place all these mechanical forces encountered by cancer cells at the primary tumor site as well as at the invasion sites. In a second part the authors describe how these physical forces act on MIC activity and in particular of Piezo1/2, TRPC1, TRPC5, TRPM2, TRPM4, TRPM7, TRPV2, and TRPV4 and affect cancer metastasis.

This Research Topic of reviews nicely shows that understanding the pathophysiological importance of mechanosignaling and the role played by MICs therein offers the potential to design concepts of targeted “mechano-therapy”.

